# Quantification of Small‐Scale Spatial Patterns in Alpine–Treeline Ecotones

**DOI:** 10.1002/ece3.71186

**Published:** 2025-05-07

**Authors:** Lukas Flinspach, Thorsten Wiegand, J. Julio Camarero, Enric Batllori, Emilia Gutiérrez, Maaike Y. Bader

**Affiliations:** ^1^ Helmholtz Center for Environmental Research Leipzig Germany; ^2^ Faculty of Geography, Ecological Plant Geography University of Marburg Marburg Germany; ^3^ Instituto Pirenaico de Ecología (IPE‐CSIC) Zaragoza Spain; ^4^ Departament de Biologia Evolutiva, Ecologia i Ciències Ambientals, Facultat de Biologia Universitat de Barcelona (UB) Barcelona Spain; ^5^ Institut de Recerca de la Biodiversitat (IRBio) Universitat de Barcelona (UB) Barcelona Spain; ^6^ Faculty of Biology, Ecology University of Barcelona Barcelona Spain

**Keywords:** agent‐based modelling, alpine treeline, pattern‐process relationships, summary statistics

## Abstract

Alpine treeline ecotones, when viewed up close, display considerable variation in spatial patterns, which have been associated with different responses to climate change. Two important dimensions of treeline‐ecotone spatial patterns are the abruptness of the change in tree height (“abrupt” vs. “gradual”) and the change in canopy cover (“discrete” vs. “diffuse”) when moving from closed forest to treeless alpine vegetation. These dimensions are suited to classify treeline ecotones into different types of patterns, but this is typically done intuitively without explicitly stated criteria, and patterns are not quantified. Consistent, robust metrics allowing comparisons between sites are lacking. We suggest several metrics to quantify abruptness and discreteness of treeline ecotones and describe how to derive these metrics from point‐pattern data of tree positions and sizes, and from high‐resolution treecover data. We developed these based on field data from the Spanish Pyrenees and an extensive dataset of treeline patterns created by the individual‐based Spatial Treeline‐Ecotone Model (STEM). We quantified the abruptness of a treeline by the largest change in canopy height, determined in 5‐m bands, between the top of the ecotone (i.e., alpine vegetation) and the first band where canopy height exceeds 3 m. We quantified the discreteness by the steepness of a logistic function fitted to tree cover. Band widths and cut‐off values were optimised for our data. Although they can be flexibly adjusted to specific case studies, standard settings are recommended to assure comparability. Our results indicate that the “discreteness” metric provides a satisfactory quantification of this pattern dimension within the dataset used here, whereas the “abruptness” pattern dimension turned out to be more difficult to capture. The metrics developed here may provide field researchers with a tool to compare their field sites in a standardised way, and potentially promote synthesis on treeline data and dynamics on a global scale.

## Graphical abstract

Spatial patterns at treeline ecotones are important indicators for potential reponses to climate change, but are difficult to define and quantify. We develop several metrics measuring “abruptness”, that is, change of canopy height, and “discreteness”, i.e., change of forest cover along the elevational gradient, using data from four field sites in the Spanish Pyrenees, and a large dataset of simulated treeline produced by the individual‐based model STEM. These metrics can help synthesise global data and provide basis for international consensus on treeline patterns.

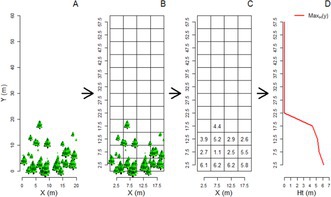

## 1 Introduction

As a distinct ecological boundary, the alpine treeline ecotone has drawn considerable attention of geographers and ecologists alike (Holtmeier and Broll 2007; Körner 1998; Malanson et al. 2007, 2011; Stevens and Fox 1991). Globally, growing‐season mean temperature and length appear to set a hard limit to the expansion of forests (Körner and Paulsen 2004; Paulsen and Körner 2014). This relation to temperature makes the alpine treeline interesting for climate change research (Harsch et al. 2009; Smith et al. 2009). Indeed, infilling of the treeline ecotone as well as upward shifts of the treeline have been reported from mountain ranges globally, even if these responses are far from universal (Batllori and Gutiérrez 2008; Birre et al. 2023; Díaz‐Varela et al. 2010; Harsch et al. 2009; Harsch and Bader 2011; Lu et al. 2021; Sigdel et al. 2024). On closer inspection, the alpine‐treeline ecotone (hereafter “treeline ecotone”), which includes the entire transition zone from closed upper‐montane forest to treeless alpine vegetation (Holtmeier 2009), shows a variety of patterns in the transitions of tree height and density (Bader et al. 2021).

**FIGURE 1:**
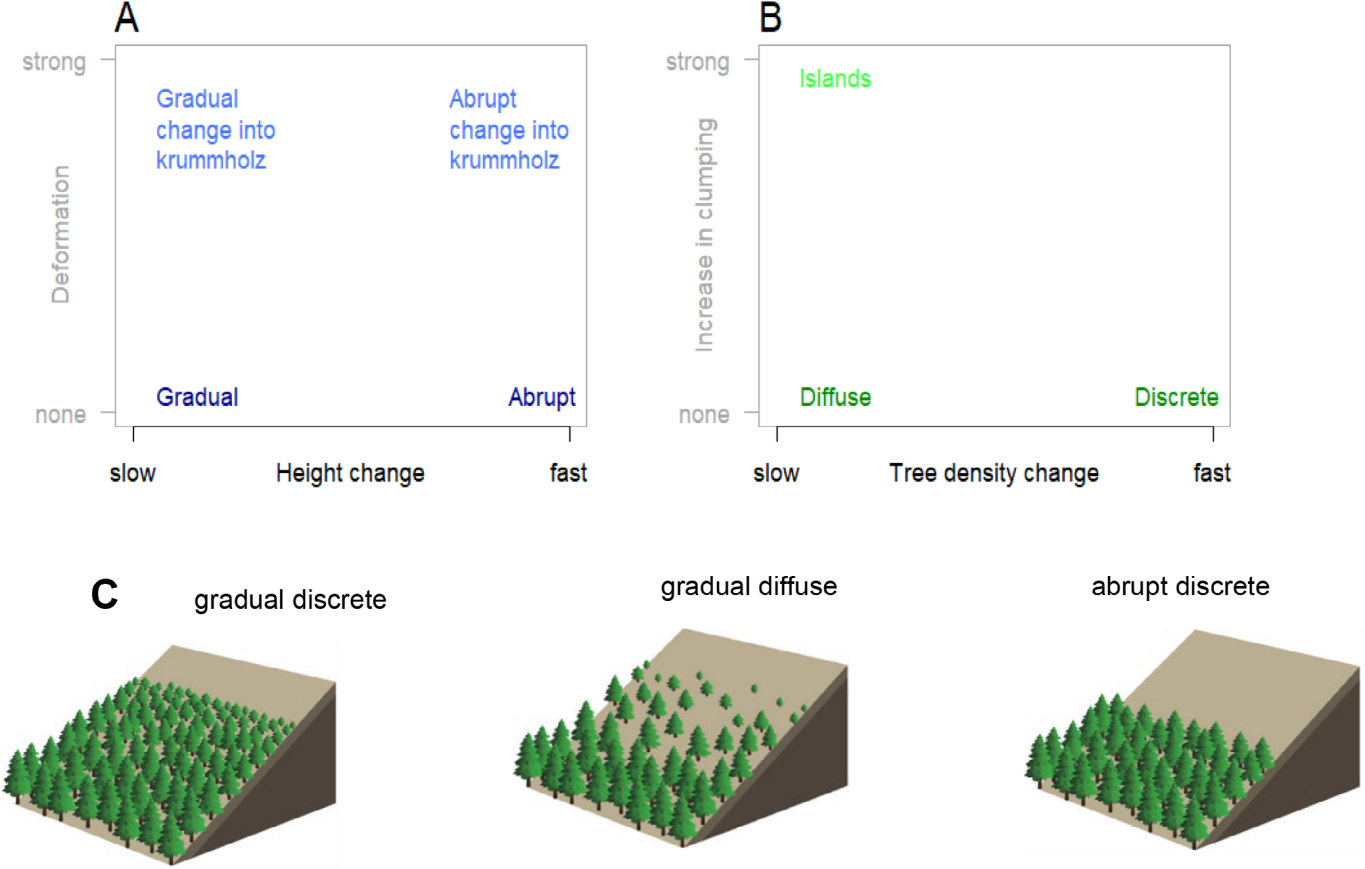
The two perspectives on treeline ecotones (lateral, A, and top‐down, B) and their two pattern dimensions (adapted from Bader et al. 2021). (A) From a side‐ways perspective, tall, single‐stemmed trees can be replaced suddenly by low‐stature alpine vegetation (“abrupt” treelines) or by stunted, multi‐stemmed krummholz (“abrupt krummholz treeline”), or the tree‐height transition can be slow and continuous (“gradual”), possibly additionally involving stunting (“gradual change into krummholz”). (B) Seen from above, the forest may approach the alpine vegetation as a closed front (“discrete”) or slowly open up. In the latter case, there may an increase in tree clustering (“islands”), or not (“diffuse”). (C) Examples of treeline forms: Gradual discrete, gradual diffuse, and abrupt discrete. Taken from Bader et al. (2021).

Interestingly, there appears to be a relationship between recent treeline ecotone dynamics, interpretable as their sensitivity to climate change, and the spatial structure of these ecotones (Elliott 2011; Hansson et al. 2021; Harsch et al. 2009; Lu et al. 2021). This relationship implies the potential of spatial pattern to act as an indicator of future dynamics, which is important for planning climate‐change adaptation. However, these relationships are not yet understood sufficiently to optimally use this indicator function. One of the reasons for this lack of understanding is that until recently, no consistent terminology about these spatial patterns was available. Hence, to improve communication and interpretation of these patterns, Bader et al. (2021) developed a framework that presents these patterns as four axes of variation along which treelines can be placed (Figure 1).

Seen from a lateral perspective, a change in tree size from forest to treeless vegetation (*x*‐axis Figure 1A) may be sudden and step‐like (abrupt), or involve a slow and continuous height decline (gradual). In addition to getting smaller, at some sites, trees might also get increasingly stunted and damaged, forming “krummholz” when damage reduces them to growth forms below tree size (*y*‐axis Figure 1A). Seen from above, the transition of tree cover and density from closed forest to alpine vegetation (*x*‐axis Figure 1B) may be sudden (discrete), when the forest approaches the alpine in a closed front, or slow (diffuse), when the canopy slowly opens up into sparse cover at the treeline. The latter may coincide with an increase in tree clustering (island formation, *y*‐axis Figure 1B). The distinction of these two perspectives is especially important since they reflect different processes: height growth and biomass loss (lateral perspective) or establishment and horizontal growth (top‐down perspective). Previous studies have tended to merge the terms “abrupt” and “discrete” on the one, and “gradual” and “diffuse” on the other hand (e.g., Armand 1992; Dearborn and Danby 2020; Greenwood et al. 2014; Lu et al. 2021). If it is not clearly defined what is meant by these terms, it is hard to imagine what such an ecotone really looks like or to relate such patterns to processes, which hampers further synthesis (Bader et al. 2021).

The treeline‐pattern framework can be summarised in 12 conceptual forms of treeline ecotones, which are located at the extremes of the suggested pattern axes (Figure 1A,B). It can be expected that these forms appear under different limiting processes, and/or at different points in time along trajectories of change. Different patterns result from differences in the demographic processes of the involved tree populations, which depend on vegetation composition and climate conditions, but which can also be modified by land use (Batllori and Gutiérrez 2008; Miehe and Miehe 1994), natural disturbances (Cairns and Moen 2004) or geomorphological constraints (Malanson et al. 2007; Resler 2006; Resler et al. 2005). In the context of the treeline‐pattern framework of Bader et al. (2021), the spatial scale of the treeline forms is defined as the hillslope scale, that is, a transect through an ecotone about 50 m wide on a homogenous slope. Due to variation in topography and disturbance regimes, different patterns may occur within the same mountain, pointing to differences in local conditions and demographic processes.

Although the distinction of treeline forms is intuitive (e.g., Figure 1c), it is often hard to assign a given treeline to one specific form, both directly in the field and based on field or remotely‐sensed data. For field sites, researchers usually identify these spatial patterns by intuition and experience, taking the entire ecotone and the context on the slope into account. Objective criteria are rarely stated explicitly, even when sites with contrasting patterns are directly compared (e.g., Armand 1992; Cieraad and McGlone 2014; Lu et al. 2021). This has to do, on the one hand, with the variability in form along some treeline ecotones, and on the other hand with a lack of pattern metrics and quantitative criteria for defining different pattern types. Although such criteria may appear conceptually simple at first glance, it is not a trivial task to define rapid and slow decreases in tree height (to differentiate between abrupt and gradual treelines) or to weigh the contribution of individual trees to the overall pattern. In addition, a few issues, such as canopy gaps or large height differences within the montane forest may disrupt an automated classification scheme and require a more elaborated approach to come up with suitable measures of abruptness (Bader et al. 2007). However, due to the lack of quantitative criteria, intuitive classifications by different researchers cannot be compared and meta‐analyses have had to rely on coarse and loosely defined categories (Hansson et al. 2021; Harsch et al. 2009). In addition, a manual classification becomes impracticable when many sites (e.g., studied by remote sensing, Birre et al. 2023), or modelling results are involved, so that objective, computer‐readable criteria are needed.

Metrics that summarise thematic, spatial or temporal patterns are called summary statistics. They reduce the dimensionality of the data while, ideally, minimising the loss of information. Such a dimension reduction is often necessary for analysing relationships and recognising similarities between patterns. In modelling contexts, summary statistics are essential for common methods of model validation, e.g., for sensitivity analysis or inverse model parameterization (Hartig et al. 2012, 2011; ten Broeke et al. 2016; Van Oijen et al. 2005). Examples of summary statistics frequently used for field‐based and model‐based forest data are basal area (e.g., Dislich et al. 2009), stem density, or species number or density (Köhler and Huth 2007). These summary statistics typically aggregate information across the entire area. However, treeline ecotones are spatially heterogeneous by definition, and the type and rate of change with elevation is the most important criterion to characterise the ecotone (e.g., Bader et al. 2007; Batllori and Gutiérrez 2008; Wiegand et al. 2006). Some other characteristic patterns, such as presence of krummholz or islands, occur only in the upper parts of the ecotone and their detection thus also requires spatially differentiated summary statistics (Bader et al. 2021).

Even within alpine treeline research, few studies have focused on gradient‐based pattern analysis, and those that have done it lack the ability to quantify patterns across different types of ecotones. For example, Wiegand et al. (2006) used data vectors tracing mean height and density across the elevational axis of their field transects and extracted higher‐level indices from these. Although effective in their application, their metric based on mean tree height is sensitive to the presence of subcanopy trees, which are uncommon in their *Pinus uncinata* forests but may be more important in other treeline ecotones (Batllori et al. 2010). In a different approach to quantify treeline patterns directly, Bader et al. (2007) fitted a logistic function to canopy‐height data of 50 tropical treeline‐ecotone transects, using the distance along the transect as explanatory variable. From the fitted function parameters, they inferred the abruptness of the ecotones. However, the approach was not robust to gaps in the canopy, and had to be backed up by a manual site‐by‐site assessment of the data. This introduces subjectivity and would be impossible to do for large datasets based on remote sensing or model output. Batllori and Gutiérrez (2008) fitted linear and spline functions to describe smooth and step‐like transitions in tree age and height at 12 sites in the Spanish Pyrenees. Buckley et al. (2016a) applied codispersion analysis to detect anisotropic aggregation and segregation in spatial data, which could be used to identify island formation at treelines (Buckley et al. 2016b). Their method relies on spatially random null models, which may be impractical to compute for large datasets. Similarly, Dearborn and Danby (2020) use a transformation of Ripley's *K*(t) and null models to quantify diffuse, island and discrete treeline forms. Therefore, to date, replicable metrics to quantify treeline‐ecotone patterns across ecological contexts and compatible with the output of individual‐based treeline tree population models are lacking.

Here we aim to develop a systematic approach to quantify spatial ecotone patterns within the context of the conceptual framework presented by Bader et al. (2021). Our new metrics are applicable to both modelled and field‐sampled data in alpine treeline ecotones, while remotely‐sensed patterns of tree cover, preferably at sub‐meter spatial resolution could also be used for the discreteness metrics. The approach works best for a transect width of about 50 m, and can hence be used to distinguish and discuss different patterns occurring within a mountain or mountain range. We concentrate on the two most challenging pattern dimensions: the abruptness (opposite of gradualness) and discreteness (opposite of diffuseness or level of thinning from closed forest into islands, Figure 1). Methods and metrics describing trends in the level of clustering (or “islandness”) along ecotones are described elsewhere (Buckley et al. 2016b) and will not be discussed here, although we do propose a simplified approach in the Appendix (Section A.14). Likewise, although the prevalence of a krummholz zone at a treeline ecotone is a characteristic holding much information about environmental drivers of the ecotone structure, we leave quantification of krummholz patterns to future studies, but suggest a method to approach this in the Appendix (Section A.13, Figure A12). In this study, we developed and tested the treeline pattern metrics and classification based on simulated data from the individual‐based Spatial Treeline Ecotone Model (STEM). A detailed introduction and documentation of the STEM is outside the scope of the present study that focuses on development of the treeline pattern metrics. The metrics were validated and on field data from the Spanish Pyrenees (Batllori and Gutiérrez 2008; Camarero and Gutiérrez 2002). Specifically, we address three main questions:

1. Which combination of summary statistics best describe the forest structure relevant for the abruptness and discreteness of the ecotone?
2. What higher‐level metrics best describe the changes in these summary statistics along the ecotone and thus allow to allocate the treeline along the abruptness and discreteness dimensions (as shown in Figure 1 and described by Bader et al. 2021)?
3. Can we identify thresholds in these metrics that clearly delineate different treeline forms, and if so, what are these threshold values?

We aim to provide field ecologists, remote‐sensing users and modellers a helpful tool to assess spatial patterns at their ecotone research sites in a robust and consistent manner. This could allow comparison and synthesis on a global scale, allowing us to better understand and forecast treeline dynamics (Price et al. 2022).

## 2 Methods

### 2.1 Model Description

We developed our method on the basis of a large dataset of simulated treeline ecotones. The simulated data provided a wide range of treeline ecotone patterns and were found to be suitable to test the applicability and generality of different classification metrics and criteria.

The simulated dataset was produced by the Spatial Treeline Ecotone Model (STEM 1.0), which will be presented elsewhere. It is a spatially explicit, individual‐based model implemented in the Netlogo software version 6.1.1. (Wilensky 1999). A detailed model description, including an ODD protocol (Grimm et al. 2020, 2010, 2006), is provided as Supporting Information. Briefly, STEM implements the “first‐level” processes growth, mortality and dieback (Bader et al. 2021; Harsch and Bader 2011) as functions of elevation, with adjustable gradients, and includes neighbourhood interactions through the modification of these processes. The first‐level processes provide the most basic descriptions of limiting demographic processes along the treeline ecotone in dependence of environmental gradients. Mortality includes a lack of viable seeds and germination and establishment failure, whereas dieback describes loss of above‐ground biomass, for example, through wind abrasion (Harsch and Bader 2011). Facilitation in the form of increased seedling survival in the vicinity of established individuals (Batllori et al. 2009) is optional in the model. Competition in the form of growth reduction and increased mortality is always operating and regulates tree densities. Elevation is assumed to increase along the *y*‐axis of the simulated area.

### 2.2 Model Data Generation

For this study, the length of the simulated transects was set to 180 m (along the elevational gradient) and the width to 60 m. The length corresponds to the longest field site used (see below), the width was selected to minimise model computation time while assuring a sufficiently‐consistent data output. The simulations ran for 800 years, starting from an empty simulation area. This timespan was generally sufficient for the model to reach an equilibrium. Transient patterns were not included in this analysis. Although we consider the temporal trajectories of treeline patterns highly informative for indicating the limiting processes at the site, we do not expect any spatial patterns to categorically disappear from our dataset during those 800 years.

We used model simulations that showed in the lowest part of the transect realistic ranges of tree densities, cover, size and age structures (based on the of the four field sites, see Table A1) and that produced a treeline ecotone (i.e., trees disappeared at the higher end of the transect). We obtained 12,149 parameterizations for which all six conducted replicates (72,894 simulations out of 185,130 in total, see Table A2) met the specified criteria. A more detailed description of the simulation experiments and filtering processes can be found in the Appendix (Sections Bottom Forest Parameterisation, Field Data‐Based Rejection Filters, Gradient Run Simulation Experiments, Treeline Acceptance Criteria). Further processing of the model output and calculation of the metrics described below was performed using R version 4.2.2 (R Core Team 2022) and the packages “tidyr” (Wickham et al. 2023), “stringr” (Wickham 2022), “minpack.lm” (Elzhov et al. 2023), “purrr” (Wickham and Henry 2023) and “data. table” (Dowle and Srinivasan 2023).

### 2.3 Field Data

**FIGURE A1:**
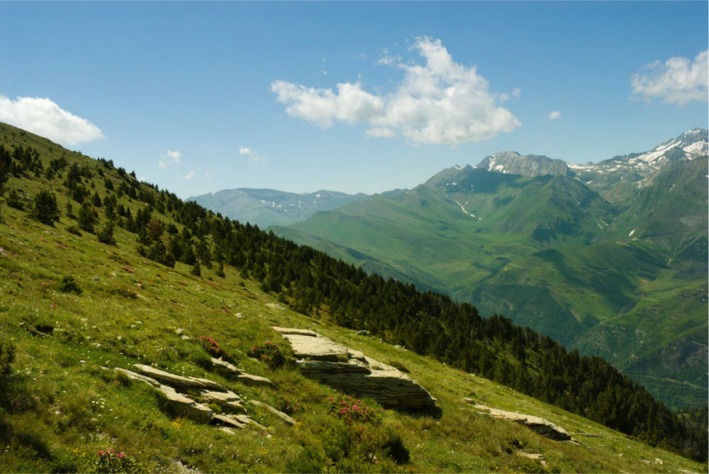
The Portell site (Photo E. Batllori).

**FIGURE A2:**
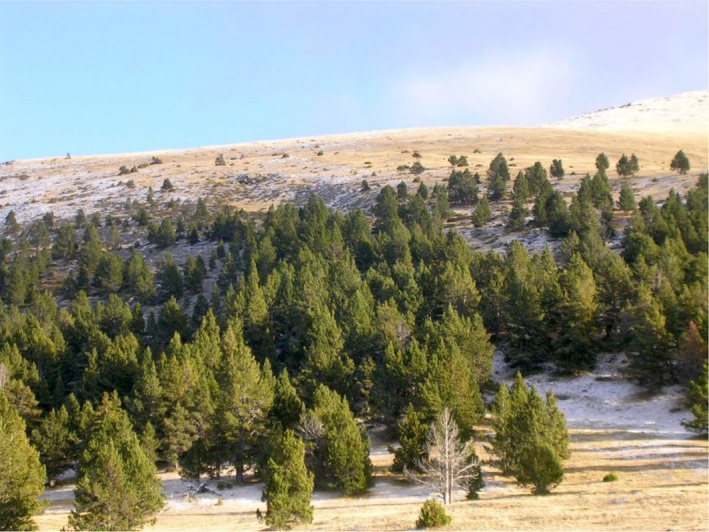
The Capifonts site (Photo E. Batllori).

**FIGURE A3:**
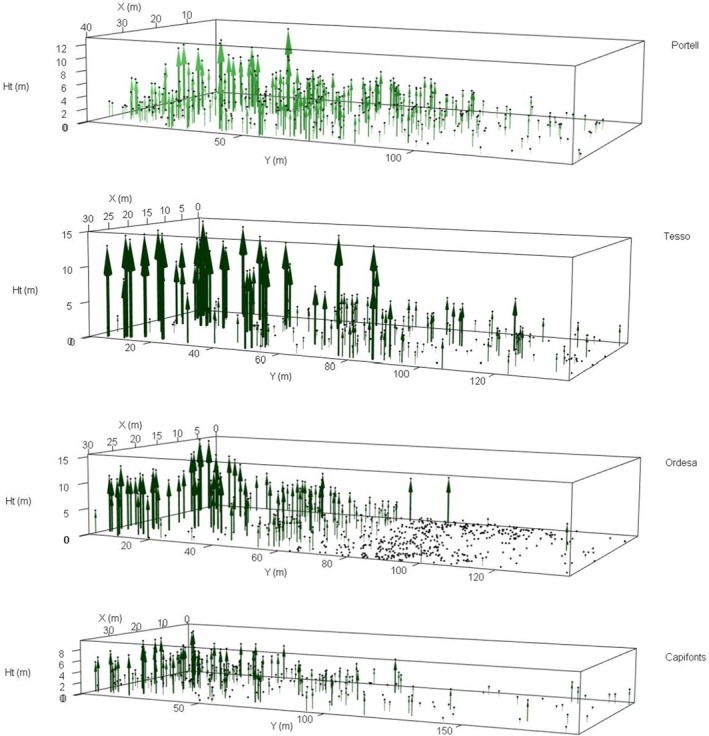
3D representations of the four field sites. Each arrow represents one tree, with arrow length indicating tree height in m. Note the wide belt of krummholz individuals with low height at the Ordesa site.

**FIGURE A4:**
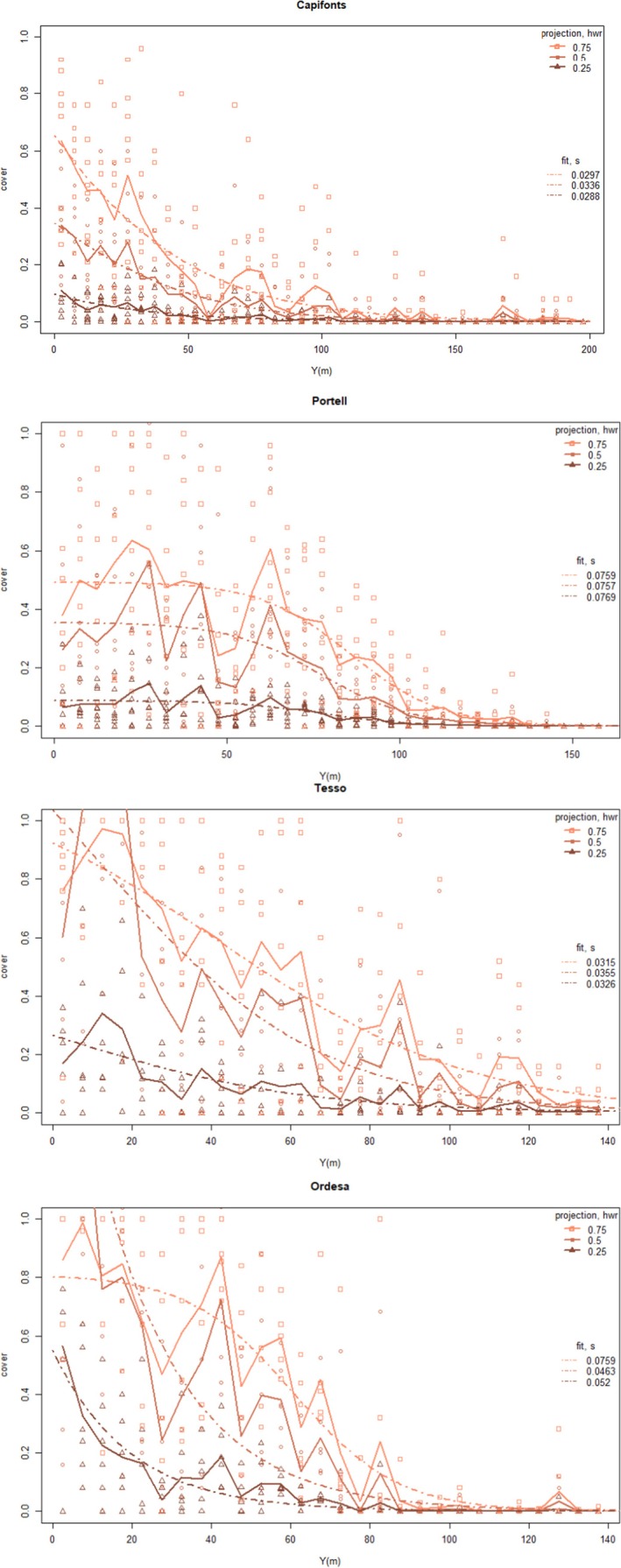
Treecover at the four field sites, assuming different ratios of tree height to crown diameter (*hwr* parameter). Only at the Ordesa site the parameter affects the cover fit, and the discreteness metric s. However, the classification of the site as diffuse (*s* < 0.2) is not affected. For the calculation of s see main text, section “Discreteness metric”.

The four field sites used here (Capifonts and Portell: Batllori and Gutiérrez 2008; Tesso, Ordesa: Camarero and Gutiérrez 2002) are located in the Spanish Pyrenees. A visualisation of the field sites is presented in the Figures A1, A2, A3. The plots sampled at these sites are 30‐40 m wide and 140‐180 m long. The contrast between ecotone patterns is largest between Ordesa and Tesso, with Capifonts and Portell ranging between them (Martinez et al. 2011). The treeline ecotone at Ordesa is considered to be an abrupt diffuse krummholz treeline (Camarero and Gutiérrez 2002). At Tesso, by contrast, the ecotone hardly has any krummholz individuals (Camarero and Gutiérrez 2002) and is described as gradual diffuse (Martinez et al. 2011). Capifonts and Portell are described as intermediate. Capifonts has a small krummholz belt. It can be described as a (gradual) diffuse krummholz treeline. The Portell treeline is considered disturbed, as it displays a step‐like transition in mean tree age within the ecotone. This is probably a remnant of former pastoral use, which is likely to have distorted the natural patterns at the site (Batllori et al. 2010). None of the four field sites are discrete. As cover or crown diameter data were not available for all of the field sites, we assumed crown diameter to be ½ of tree height, which matches the height‐to‐width ratio in the model data. This assumption is a simplification and not based on allometric data. We tested the values 0.25 and 0.75, however, these changes influenced the discreteness metric (see below) at only one of the four sites, and did not change the classification of the site, see Figure A4.

### 2.4 Calculation of Subplot Summary Statistics

**FIGURE 2:**
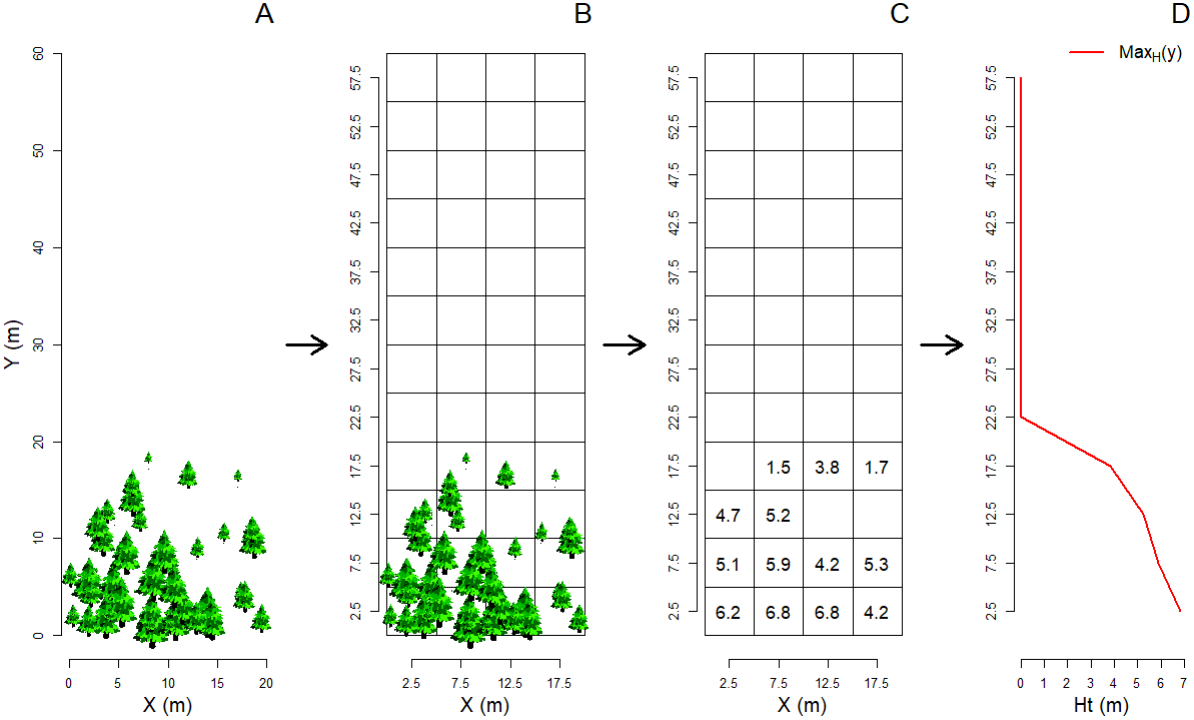
Schematic representation of the data vector generation for describing treeline‐ecotone patterns, using maximum canopy height as an example: Point‐pattern data (A) are grouped according to a grid composed of elevational belts divided horizontally into subplots (B; note that in most cases the number of belts and subplots will be larger than in this illustration). The summary statistics, for example maximum tree height, are calculated for each subplot (C). Subplot summary statistics are aggregated into belt‐wise data vectors (D), for example by using the highest or second‐highest value of subplot‐level maximum tree height within a given belt. This results in the Max_H_ (y) or Max2_H_(y) summary statistic vector.

The primary model output consists of individual point‐pattern information on all living trees and seedlings at the end of the simulation, including their coordinates, crown diameter, height and age. Several steps were required to reduce the dimensionality of this dataset into single, scalar metrics that could be used for further model analysis (Hartig et al. 2011). We divided the simulated or field area into several elevational belts (i.e., horizontal strips perpendicular to the slope), which were additionally divided into subplots of 5 × 5 m (Figure 2B). The width of the horizontal belts corresponds to the resolution of the data vectors. For the simulated data, this resulted in *n*_b_ = 36 belts and 12 square subplots per belt. For the field data, we applied the same method. As the field transects were narrower, the number of subplots available was lower (8 per belt for Portell, Capifonts, Batllori and Gutiérrez 2008; 6 for Ordessa, Tesso, Camarero and Gutiérrez 2002).

The different summary statistics were first calculated for each subplot individually (Figure 2C). These statistics described the mean tree cover *C* and densities *D* of individuals larger than 0.5 m, and maximum tree height Ht. For tree height and density, these summary statistics were subsequently aggregated to belt‐wise values (Figure 2D), while for tree cover a function was fitted through the mean values at the subplot level. This beltwise‐aggregation approach produced output vectors containing summary statistics as a function of distance along the transect, representing increasing elevation (*y*: the distance of the middle of the elevational belt to the bottom of the simulated area, Figure 2D), and is similar to the approaches used by Wiegand et al. (2006) and Martinez et al. (2011).

We iteratively expanded and adapted these data vectors and scalar metrics to better capture the patterns at exemplified ideal treeline forms. We then applied the metrics to our simulated and field data, identified limitations, and added further adjustments to create a work flow and metric definitions that best captured the different pattern dimensions in most of the examples. The final selected metrics and their applications are explained in more detail in the following sections, while the application of these metrics to our datasets and some suggestions for threshold values for defining treeline forms are presented in the results section.

### 2.5 Abruptness Metrics

**FIGURE 3:**
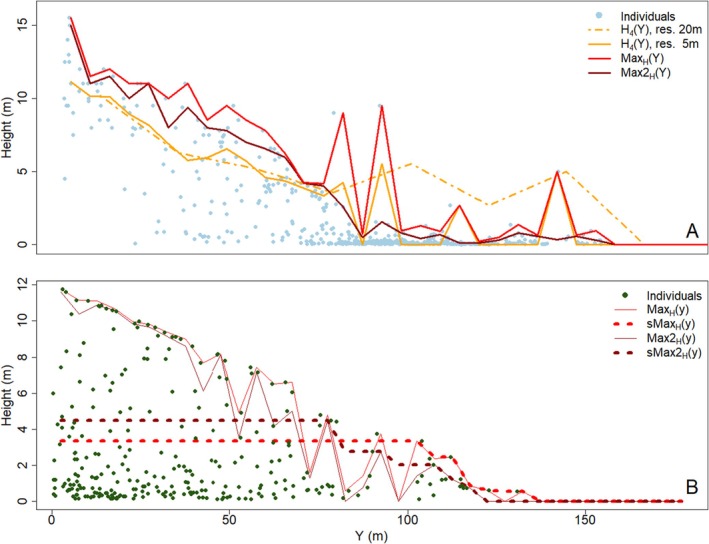
Illustration of canopy height data vectors (A, B). (A) Different data vectors quantifying the canopy height pattern at the Ordesa field site. *Max*_*H*_(*y*) (red) traces the tallest individual in the belt, *Max2*_*H*_(*y*) (dark red) traces the second‐highest subplot maximum. The average height of larger trees (here > 1.5 m; layer 4 in the model output, *H*_4_(*y*)) at 5 m (orange solid line) and 20 m resolution (orange dotted line) shows less variation, but does not capture the real canopy height. (B) Example of a treeline ecotone with gaps in the canopy. Truncation and smoothing helps clearing the pattern and removes gaps that could lead to patterns being mistakenly held for abrupt declines (dotted lines).

The first decision is the choice of an appropriate data vector for reducing the dimensionality of the individual‐based data. Previous authors (Martinez et al. 2011; Wiegand et al. 2006) calculated the average height of all trees (excluding krummholz) in each subplot, and derived data vectors from these averages. While these summary statistic vectors are generally able to detect rapid transitions in tree height, they give a strong weight to subcanopy trees. Since the tallest trees demonstrate the growth potential of the elevational belt, and not the smaller ones, which may be small due to a younger age or competition, we decided to use summary statistic vectors that give more weight to the tallest trees (Figure 3A).

**FIGURE A5:**
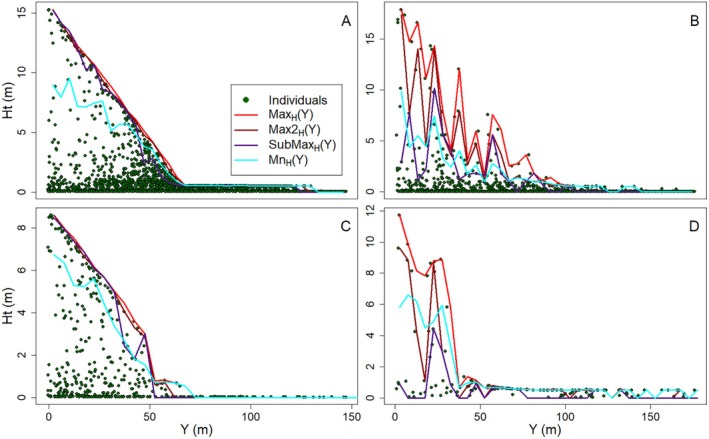
Visualisation of the tested canopy height data vectors in four simulated treelines. Max_H_ (y) (red) traces the tallest individual in each belt, Max2_H_ (y) (brown) the second‐tallest subplot canopy in each belt. SubMax_H_ (Y) (violet) traces the maximum canopy height in each belt after the tallest individuals of each suplot were removed. Mn_H_ (Y) describes the average subplot canopy height.

We therefore defined subplot canopy height as the height of the tallest individual within the subplot, or NA if there were no individuals taller than 0.5 m. From the subplot canopy‐height data, we derived two data vectors: (i) *Max*_*H*_(*y*) is the highest subplot canopy height in each elevational belt *y* (i.e., the height of the tallest individual within each belt), set to 0 if there is no individual taller than 0.5 m in the entire belt. (ii) *Max2*_*H*_(*y*) is the second‐highest subplot canopy height in each belt, set to 0 if there is no more than one subplot with individuals taller than 0.5 m. As the simulated area in the modelled data contained 12 subplots per belt, *Max2*_*H*_(*y*) corresponded to the belt‐wise 92‐percentile of subplot canopy height. We also tested other data vectors but they were rejected, as they performed worse in tracing canopy height (see Figure A5).

As the distinction between abrupt and gradual treelines concerns the transition from the uppermost tall trees to alpine vegetation, and not any canopy height differences, gaps or lower‐stature stands within the forest, we truncated the canopy data vectors *Max*_*H*_(*y*) and *Max2*_*H*_(*y*) to excluded height variation within the forest. In practice, descending from the top of the transect, we registered the first (i.e., highest‐elevation) belt with trees above 3 m (=*Max*_*H*_(*y*_t_) in truncation belt *y*_t_) and set all subsequent values (from lower belts) to that height (Figure 3B). The resulting truncated data vectors are called *tMax*_*H*_(*y*) and *tMax2*_*H*_(*y*), respectively. Thus, we first identified the belt *y*_t_ of the topmost belt with trees > 3 m by the condition.

(1) $${\mathrm{Max}}_{H}\left(y_{t}\right)>3\;m\;\text{and}\;{\mathrm{Max}}_{H}\left(y\right)<3\;m\;\text{for}\;y=y_{t+1}\ldots n_{b}.$$

The truncated data vector *tMax*_*H*_(*y*) is then given by.

(2) $${\text{tMax}}_{H}\left(y\right)={\mathrm{Max}}_{H}\left(y_{t}\right)\;\text{if}\;y\leq y_{t}\;\text{and}\;{\text{tMax}}_{H}\left(y\right)={\mathrm{Max}}_{H}\left(y\right)\;\text{otherwise}.$$

The same procedure applies for the second‐highest subplot canopy height *tMax2*_*H*_(*y*).

Finally, we smoothed these vectors. Descending from the topmost belt, we iteratively replaced each value of *tMax*_*H*_(*y*) and *tMax2*_*H*_(*y*) with the value of its upslope neighbour belt if that value was higher, until we reached the belt *y*_t_. This procedure removed gaps within the low‐stature canopy and allowed for clearer distinction of gradual vs. abrupt treelines (Figure 3B). The resulting smoothed data vectors are called *sMax*_*H*_(*y*) and *sMax2*_*H*_(*y*).

To detect potentially abrupt declines of canopy height, we calculated the difference in the smoothed canopy height vectors between two successive belts.

(3a) $${\text{δMax}}_{H}\left(y\right)=\left[{\text{sMax}}_{H}\left(y+1\right)–{\text{sMax}}_{H}\left(y\right)\right]$$

(3b) $${\text{δMax2}}_{H}\left(y\right)=\left[{\text{sMax2}}_{H}\left(y+1\right)–{\text{sMax2}}_{H}\left(y\right)\right]$$

These procedures express canopy‐height differences in absolute terms, but for characterising the discontinuity in height decline at the forest edge, the decline relative to the overall forest stature may be of more interest. We therefore normalised these data vectors with the largest overall canopy heights in the entire transect:

(3c) $${\text{nδMax}}_{H}\left(y\right)=\frac{{\text{δMax}}_{H}\left(y\right)\;}{\;\max\left[{\mathrm{Max}}_{H}\left(y\right)\right]}\;\text{for}\;y=1,\ldots \mathrm{nb}-1$$

(3d) $${\text{nδMax2}}_{H}\left(y\right)=\frac{\text{δMax}2_{H}\left(y\right)\;}{\;\max\left[\mathrm{Max}2_{H}\left(y\right)\right]}\;\text{for}\;y=1,\ldots \mathrm{nb}-1$$

Note that the values of these differences will generally be negative because they describe a decline in canopy height. We then considered the lowest value of the latter two data vectors, that is, the largest decline of canopy height between two successive belts, normalised to the interval [0, 1], as final measures of the abruptness of the treeline ecotone. However, we switch the sign here for more intuitive use and consistency with the other metrics, where higher values correspond to stronger expression of the pattern:

(4a) $$a\_\mathrm{abr}=-\min\left[{\text{δMax}}_{H}\left(y\right),y\right]\;\text{absolute abruptness}\;1$$

(4b) $$a\_\text{abr2}=-\min\left[{\text{δMax2}}_{H}\left(y\right),y\right]\;\text{absolute abruptness}\;2$$

(4c) $$n\_\mathrm{abr}=-\min\left[{\text{nδMax}}_{H}\left(y\right),y\right]\;\text{normalised abruptness}\;1$$

(4d) $$n\_\text{abr2}=-\min\left[{\text{nδMax2}}_{H}\left(y\right),y\right]\;\text{normalised abruptness}\;2$$

High values of the absolute abruptness metrics a_abr and a_abr2 mean that the transition from trees > 3 m to alpine vegetation (possibly with seedlings  < 0.5 m) involves a large difference in tree height between two adjacent belts, indicating an abrupt treeline. This value can be higher at the edge of forests with higher trees. In contrast, for the normalised abruptness metrics n_abr and n_abr2 high values mean that a large proportion of forest height is gained in only one step, also indicating an abrupt treeline. This value is likely to be higher at the edge of short‐statured than tall forest. By calculating and reporting both metrics, different types of comparisons between sites are possible. Table 1 lists the metrics with a brief description.

**TABLE 1:** Summary of pattern metrics for characterising alpine‐treeline ecotones. Additional suggested metrics for krummholz and islands can be found in the Table A3.

Abruptness	a_abr	Largest decline in smoothed truncated maximum belt‐level canopy height (in m)
	a_abr2	Largest decline in smoothed truncated second‐largest subplot‐level canopy height per belt (in m)
	n_abr	Largest decline in smoothed truncated maximum belt‐level canopy height divided by the max transect canopy height (dimensionless)
	n_abr2	Largest decline in smoothed truncated second‐largest subplot‐level canopy height per belt divided by the max transect canopy height (dimensionless)
Discreteness	s	Steepness of the logistic function fitted to the cover data vector, indicating the rate of density change along the ecotone

### 2.6 Discreteness Metrics

**FIGURE 4:**
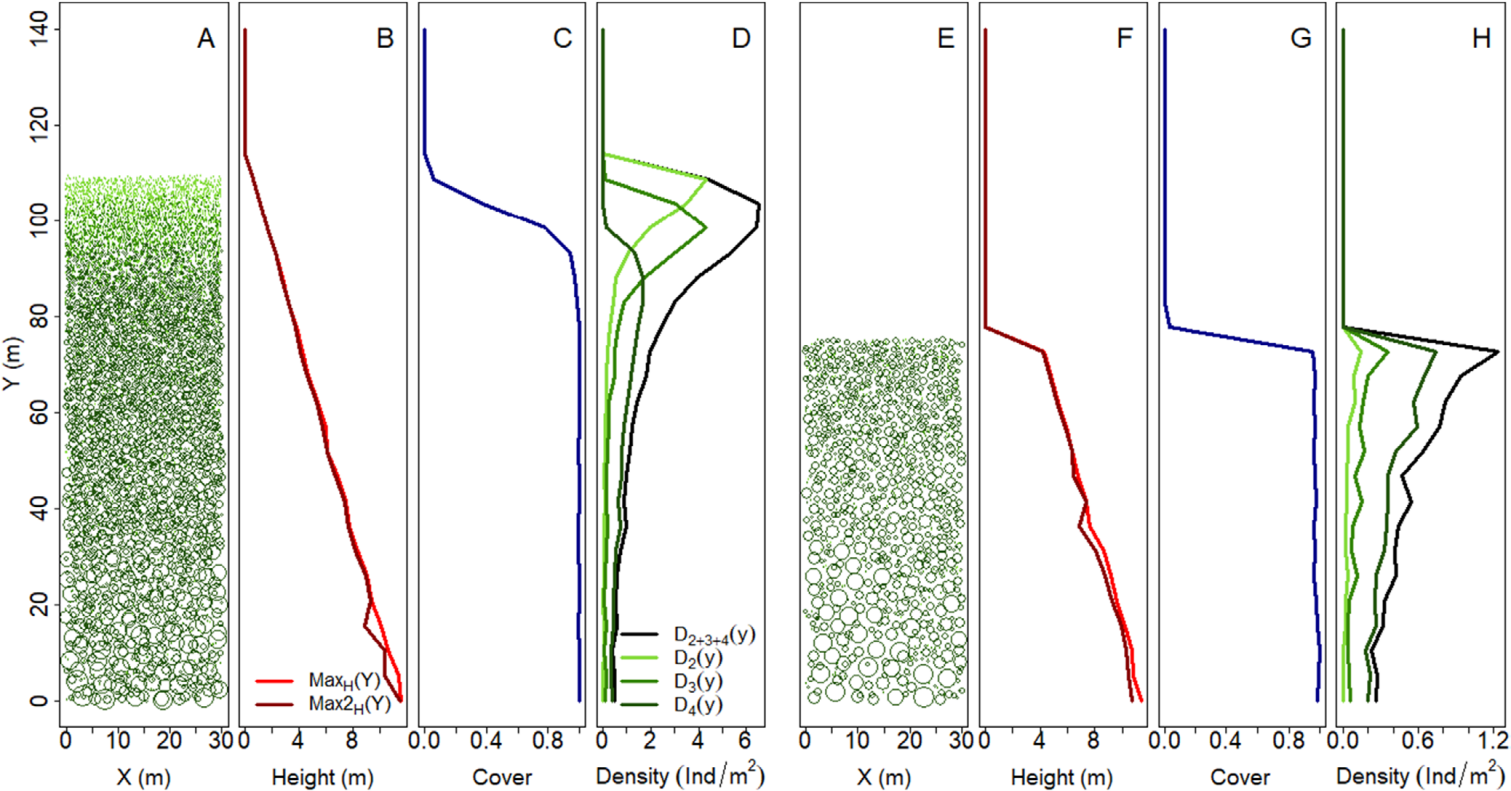
Examples of an idealised gradual discrete (A–D) and abrupt discrete treeline ecotone (E–H). (A) Top‐down perspective; symbol sizes proportional to crown radius. Colour corresponding to height classes (dark green: Layer 4, trees > 1.5 m, green: Layer 3, tree individuals between 0.5 and 1.5 m, light green: Layer 2, tree individuals between 0.1 and 0.5 m). (B) Canopy height, decreasing gradually, as shown by canopy height vectors *Max*_*H*_(*y*) and *Max2H*(*y*). (C) Tree cover, decreasing suddenly at the treeline following a sigmoidal curve, that is, forming a discrete forest edge. (D) Tree density, following a bell‐shaped curve that is cut off at the top. Subscripts here refer to height classes as in (A), following the same colour scale. For the different height classes, the peaks of these curves are shifted along the elevation axis. (E) Abrupt treeline ecotone from a top‐down perspective, colours and symbols identical to (A). (F) Tree height decrease shows a clear step, that is, the decrease is abrupt. (G) Tree cover follows a similar trajectory as in (C), showing a discrete forest edge. (H) Tree density, on the other hand, follows a different pattern: Moving uphill, it increases exponentially until the cut‐off, and for all height classes, peak densities and subsequent cut‐off are located at the same elevation.

The discreteness of a treeline as seen from above is determined by tree cover and by tree density. Tree cover and tree density change very differently along elevation as trees get smaller (Figure 4), since at similar spacing (i.e., similar stem density), smaller trees have a lower cover and will appear more spread out than large ones. We consider tree cover to be more relevant than tree density in defining treeline‐ecotone discreteness, since it more strongly determines the physiognomy and ecosystem functions of the vegetation than stem density. In addition, it is the measure most easily observed on remote‐sensing imagery, which is a more widely available data source for treeline ecotones than field‐based point pattern data. To obtain tree cover data from point pattern field data if tree cover is not recorded, an estimation of cover based on tree height and/or stem diameter can be used.

For our data, both field and modelled data, cover was calculated by projecting (assumed) circular tree crowns on 1 × 1 m grid cells and removing overlap. Cell cover therefore ranged from 0 to 1. For each subplot, the average cell cover was calculated. More detail about this estimation is provided in the Appendix (Section A.7).

We fitted a logistic function to the subplot‐level cover data along the *y* axis. For these fits, the minpack.lm package (Elzhov et al. 2023) was used:

(5) $$C\left(y\right)=\frac{C_{\max}}{1+e^{s*\left(y-y_{l}\right)}}$$

with the parameters *C*_*max*_ (i.e., cover in the forest), *s* (transition steepness) and *y*_l_ (location of the transition along the *y*‐axis in m). We use the fitted parameters to quantify discreteness. Table 1 contains a short description of the metric.

At a discrete treeline, the sudden, steep transition from more or less constant cover in the forest to low cover in the alpine zone should result in a high value of the fitted transition steepness *s*, whereas at a diffuse or island treeline, this transition is slower, resulting in a lower value of *s*.

Due to the difficulty in capturing the trajectory of tree density along the elevational axis at a discrete treeline and its interaction with tree size in a simple mathematical function (compare the examples in Figure 4D,H), we decided not to use the density data vectors for quantifying discreteness.

## 3 Results

### 3.1 Abruptness

**FIGURE 5:**
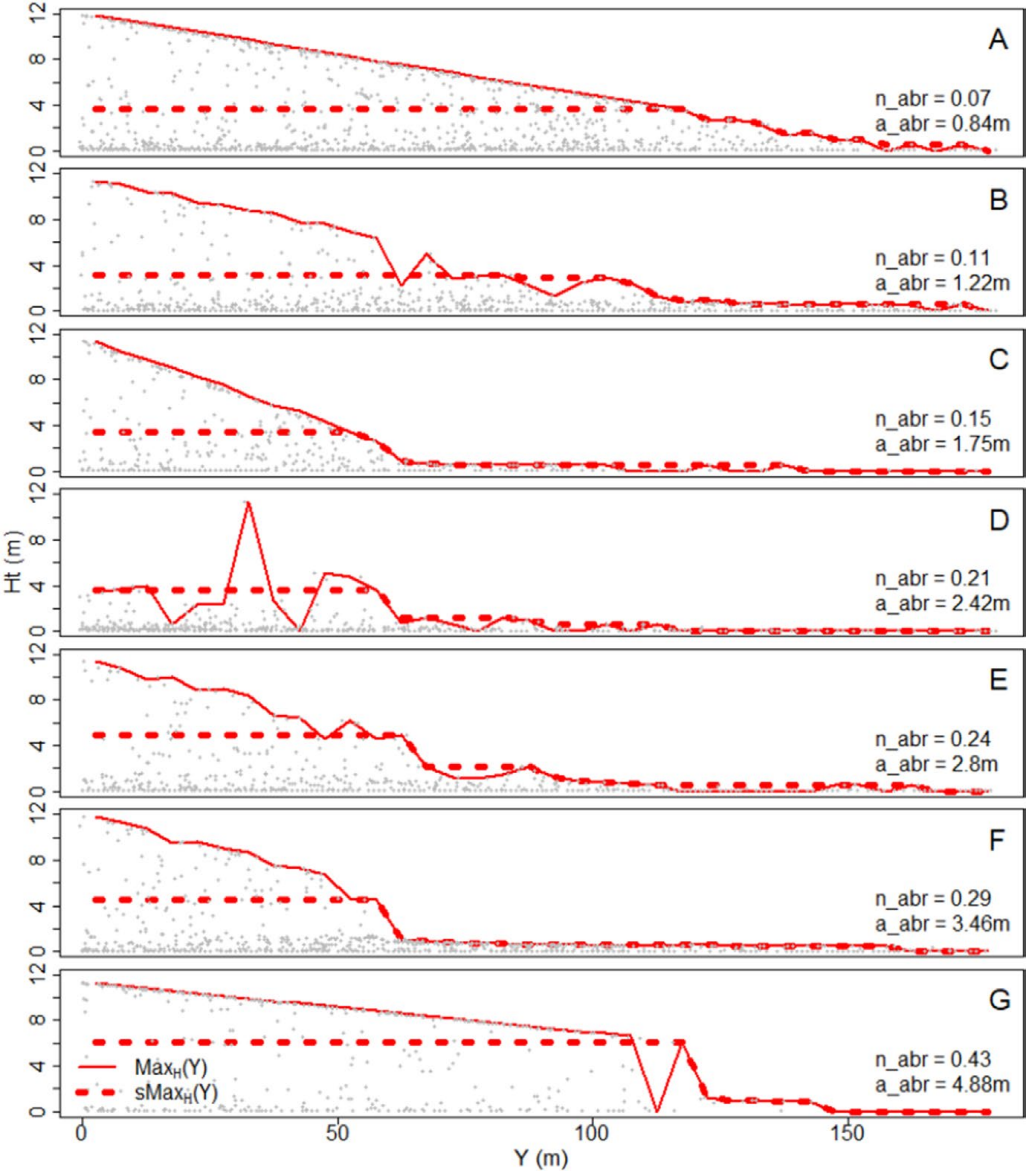
Examples of simulated treeline ecotones with increasing abruptness, as quantified by the normalised (n_abr, Equation 4c) and absolute abruptness (a_abr, Equation 4a), shown on the left. Solid lines show *Max*_H_(*y*), the height of the tallest individual within each belt, and dotted lines the smoothed truncated *sMax*_H_(*y*) (Equation 2). Grey dots indicate individuals. Given the presence of krummholz in these examples, we propose to classify treelines with n_abr  < 0.25 and a_abr  < 1 m as gradual (A), n_abr  > 0.4 and a_abr  > 2 m as abrupt (‐FG), and the remaining treelines as intermediate (‐BE). Corresponding visualisation of a_abr2 and n_abr2 can be found in the Appendix (Section A.8; Figure A6).

The quantification of abruptness by the metric n_abr (Equation 4c, 4d) yielded an ordering of datasets according to abruptness that concurred with visual inspections of the height declines across the ecotones (Figure 5).

Visual survey of our simulated dataset made clear that mid‐range values of all abruptness metrics could not be consistently assigned to either abrupt or gradual patterns, which fits the concept of these metrics representing a continuous axis, rather than a dichotomous key. Still, to allow classification into abrupt, intermediate, and gradual treelines, we propose to use a_abr > 3 m as a criterion for abrupt treelines and a_abr  < 2 m for gradual treelines (for treeline ecotones with no krummholz belt; if a krummholz belt is present, these criteria should be reduced by 1 m). For a_abr2, based on the second‐tallest subplot, the expected values are smaller than for a_abr, but the same 3‐m threshold for an abrupt treeline could be applied for a more robust assignment to the abrupt class, while for a robust assignment to the gradual class, a threshold of a_abr2 < 1.5 m could be used. For n_abr and n_abr2, we propose  < 0.25 as a criterion for gradual treelines (Figure 5E) and > 0.40 for abrupt treelines. As with a_abr2, n_abr2 can give a more robust classification as abrupt treeline at the same threshold ( < 0.40).

Applying these thresholds to the field data classifies Portell and Tesso as gradual treelines according to the normalised metrics, while the absolute metrics are inconclusive (Table 2). The other two treelines were more ambiguous (Ordesa) or intermediate (Capifonts). According to n_abr2 and a_abr2, which ignore the tallest individuals in each belt, the Ordesa site was gradual, although it was abrupt according to n_abr as well as according to our impression in the field (Table 2). This highlights the importance of considering more than one metric for describing the treeline abruptness and to manually assign the site if the metrics contradict each other.

**TABLE 2:** Abruptness classification of the four treeline ecotones in the Spanish Pyrenees with the four abruptness metrics. Treelines with n_abr  < 0.2 5 and/or a_abr  < 2 m (a_abr2  < 1.5 m) were defined as being gradual and treelines with n_abr > 0.40 and/or a_abr > 3 m abrupt.

	Field estimate	a_abr (m)	a_abr2 (m)	n_abr	n_abr2
Portell	Gradual	1.4^a^	1.5	0.10^a^	0.11^a^
Tesso	Gradual	2.9	2.0	0.19^a^	0.13^a^
Ordesa	Abrupt into krummholz	4.0^b^	1.4^c^	0.26	0.09^a^
Capifonts	Intermediate	2.8	1.9	0.29	0.20^a^

^a^ Condition for gradual treeline met.

^b^ Condition for abrupt treeline met.

^c^ Condition for gradual not met due to krummholz belt.

### 3.2 Discreteness

**FIGURE 6:**
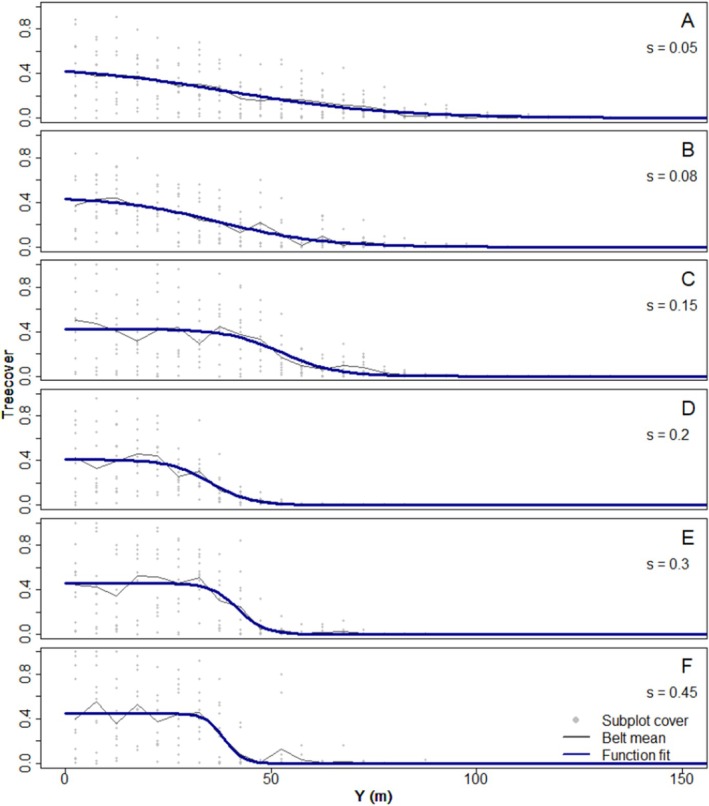
Examples of simulated treelines with increasing discreteness, as quantified by the *s* parameter (displayed right) of the fitted logistic function. Examples selected with similar y_l_ and *C*_max_. Note that this method permits some individuals to stand above the discrete edge (see I) even for rather discrete treelines. We propose to consider treelines with *s*  <  0.2 (A–C) as diffuse and *s* ≥ 0.2 (D–F) as discrete.

The discreteness metric *s* performed very well as long as there was enough forest in the lower part of the transect and enough tree‐less area towards the top to allow plateaus of high and low tree cover to be established by the logistic function (Figure 6). If the first condition was not met, the model would locate the midpoint of the transition from forest to alpine vegetation near the bottom or even below the transect (i.e., *y*_l_ ≤ 5 m). If the second condition was not met, the midpoint would be located near the top or above the transect (in our case, *y*_l_ > 180 m). We did not quantify discreteness for these treelines since a large part of the transition from forest to alpine vegetation would be located outside the transect, and discreteness can no longer be robustly estimated. A fitted *s* value > 0.5 indicates that nearly the entire transition occurs within one elevational belt. Taking horizontal irregularity of the treeline into account (i.e., sinuousity, Case and Hale 2015), we propose assigning treelines with a fitted *s* > 0.2 as discrete (Figure 6D–F). This threshold ensures the transitions spans across no more than 15 m (3 belts here). Although *s* is not directly dependent on the belt width, belts wider than 5 m will make it difficult to distinguish a truly discrete treeline.

**FIGURE A7:**
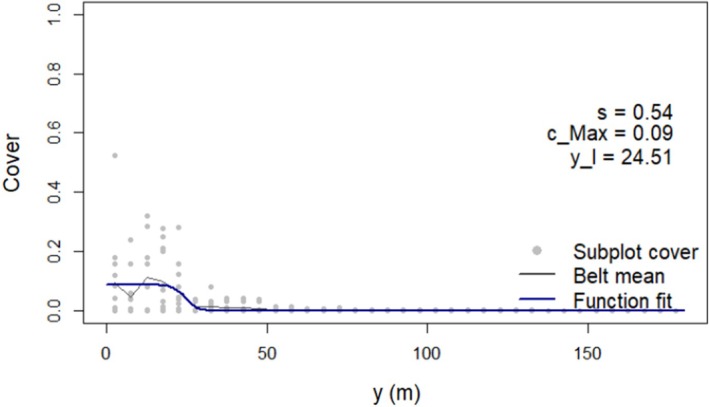
Example of a treeline with low cover. Although the transition to alpine vegetation is swift, as indicated by the high *s*, we do not consider this a discrete treeline, since there is no closed forest.

Fitting the impression of diffuseness in the field, the four field sites are not discrete according to the logistic functions fitted to the cover data, but for different reasons (Table 3, for a visualisation of the fits, see Figure A9). All had *s* values below 0.1, indicating a slow transition from closed forest to alpine vegetation. But for Capifonts and Ordesa our function fitting located the transition below or very close to the bottom of the sampled area (*y*_l_  < 5), so that *s* cannot be confidently interpreted. Portell has a fitted *C*_max_ value of 0.27, indicating a low forest cover at the bottom of the sampled area. At such low forest cover, it can be argued that the concept of discreteness is not applicable even for higher values of s (see Figure A7).

**TABLE 3:** Top‐down classification metrics of the four field sites based on tree cover. Treelines with discreteness parameter *s* larger than 0.2 are considered discrete, but treelines with low density at the lowest belt (*C*_max_ < 0.4) classified as diffuse because no discrete tree cover transition was possible here. The two sites where the midpoint of the transition was located close to the edge of the transect cannot be classified with this approach.

Site	Field estimate	*C*_max_	*y*_l_ (m)	*s*
Capifonts	Diffuse	0.80	−8.77^a^	(0.0334)
Portell	Diffuse	0.28^b^	80.26	0.0752^c^
Tesso	Diffuse	1.01	19.85	0.0316^c^
Ordesa	Diffuse	1.43	2.99^a^	(0.0347)

^a^ Not discrete because *y*_l_ ≤ 5 m or *y*_l_ > 180 m.

^b^ Not discrete because *C*_max_  < 0.4.

^c^ Condition for diffuse met.

## 4 Discussion

Quantifying and classifying treeline patterns at the hillslope scale in a robust and consistent manner is a difficult task, as variation in tree height and tree cover along the treeline ecotone can produce high variability in patterns. We developed a systematic approach to classify alpine treeline ecotones based on the abruptness of the change in tree height (i.e., abrupt vs. gradual) and the discreteness of the change in tree cover (i.e., discrete vs. diffuse), and applied it to observed treeline ecotones and treeline ecotones simulated by an individual‐based model. The pattern metrics developed here partially concurred with our subjective classification of the field sites. For example, the Portell field site was identified as gradual diffuse, which matches with expectations. However, our results also highlight challenges which may hamper automatic, standardised classification of treeline forms on the global scale in the future.

### 4.1 Abrupt to Gradual

#### 4.1.1 What Data Vector Best Captures Canopy Height?

One tall “outpost” tree individual located near the upper border of a treeline ecotone carries a substantial ecological weight. The individual has persisted at the site for a long time with few or no contemporaries, indicating that a seedling survival limitation, more than growth or survival limitation for established tree, exists at the site. This is important information for understanding the processes that determine treeline dynamics, especially since that outpost tree may act as a nucleus for further reproduction and encroachment in the future. Consequently, the data vector describing canopy height chosen here should not omit the information provided by these outposts, even if statistical intuition would guide us to weigh down the outliers. This supports the use of the Max_H_(Y) (and the derived metrics a_abr and n_abr), rather than Max2_H_(Y) or an average of tree heights.

**FIGURE A8:**
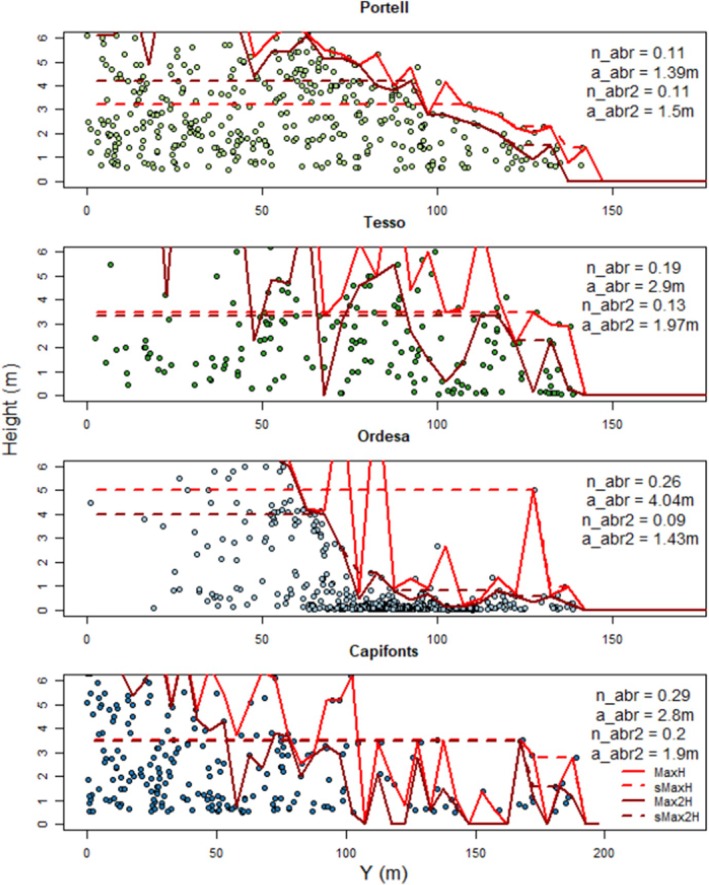
The four Pyrenean field sites, ordered by increasing abruptness according to n_abr_. Tree height is restricted to focus on the topmost trees. Note that the absolute height difference a_abr_ at the Tesso site is 2.9 m, hence bordering abruptness, even though the site is described as gradual by the field researchers. Here, the overall impression of a gradual treeline arises from the overall context of the site, including taller trees further downslope, see Figure A3. At Tesso, one 5 m high individual at Y ≈ 127 m dominates the metric and marks the site as abrupt. Ignoring the tallest individuals, the treeline at the site is gradual, according to the n_abr2_ metric. A considerable discrepancy between metrics can also be found at the Capifonts site, although the responsible individual here at approx. 170 m (2.9 m tall) appears less prominent in the context.

**FIGURE A9:**
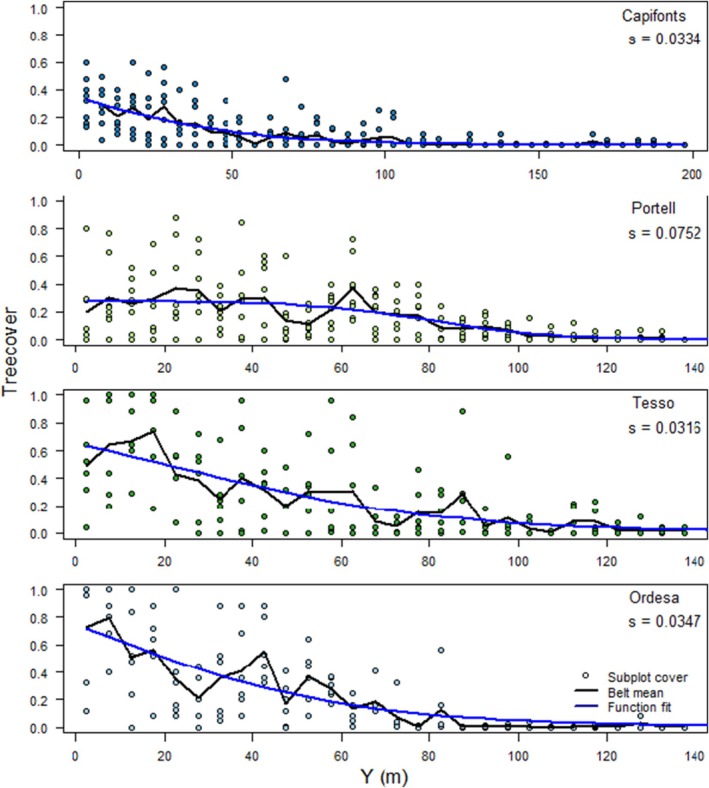
Tree cover in the four Pyrenean field s ites. Displayed is the estimated cover in each subplot (points), the estimated belt‐wide averages (black line), and the fitted logistic functions (blue). All sites show a slow transition from alpine vegetation to closed forest (s < 0.2). Hence, we consider all to be diffuse.

The Ordesa field site has previously been described as the most abrupt of the four field sites, because of the rapid transition of canopy height from the krummholz belt into the forest around *Y* = 72 m. However, at *Y* = 127 m, one individual rises about 4 m above the krummholz (see Figures A3 and A8). We argue that the abruptness of the site should be assessed based on this individual.

The Max2_H_(Y) vector and derived metrics a_abr2 and n_abr2 would remove the influence of the tallest individuals. These may be favoured at retreating treelines, where outposts could be considered relics which no longer reflect the ecological conditions at the site (see, e.g., Körner 2007).

#### 4.1.2 What Metric Best Captures the Abruptness?

Of the four pattern dimensions presented by Bader et al. (2021), abruptness is arguably the most difficult to define mathematically in a way that is robust to variation in the data. The abrupt edge may occur within, rather than at the upper edge of the ecotone, especially if krummholz is involved. Hence, it is not sufficient to consider only the height difference between the uppermost tree individuals and the alpine vegetation. We have thus selected the maximum height difference between two successive points along the canopy‐height data vector, independently of where that difference occurs on the elevational axis, as long as the lower height of the step is below 3 m and no trees taller than 3 m occur higher up along the ecotone. This criterion assures that the height difference can be assigned to the transition from forest to low‐stature alpine vegetation, or at least to very short uppermost forest or krummholz, and not to other variation in forest stature. This allowed us to apply the same approach to all treelines whether or not they have a krummholz or seedling belt in the upper section. However, it does require ensuring that gaps in the canopy do not influence abruptness.

Gaps in the canopy have also proven an obstacle in the lateral classification of treelines in a previous study, which was based on very narrow transects, so that the data was particularly sensitive to gaps (Bader et al. 2007). Since the rapid increase in canopy height below such a gap can easily be mistaken for the edge of an abrupt treeline, we decided to remove these gaps by smoothing our data vectors. The truncation, that is, the capping of canopy height once a minimum forest height of 3 m has been reached, additionally serves to remove the influence of exceptionally large individuals within the forest. The 3‐m tree height limit is also a lower limit of tree size often used for defining the treeline, although 2 m is also sometimes used (Holtmeier 2009). Like the definition of treeline spatial patterns, this quantitative definition of a tree in the context of the treeline is also arbitrary (e.g., Körner and Hoch 2023), but both are useful conventions for scientific communication and comparative research. We preferred using 3 m for calculating this metric, since a 2‐m threshold is more likely to exclude tall krummholz and other shrubs, which sometimes form a fringe preceding (looking downhill) an abrupt transition of canopy height (see Figure A11). For treelines with such fringes, truncation may remove the abrupt transition from the data vector and thus lead to misclassification. For this type of treelines, the “cutoff height” should be selected carefully and even 3 m may be too low.

**FIGURE A10:**
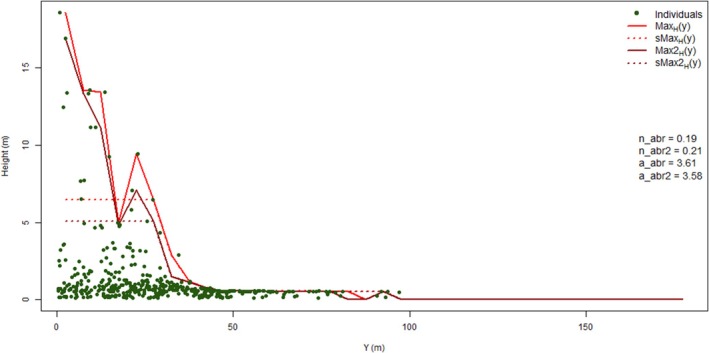
Example of a steeply declining treeline canopy. The absolute canopy height differences of a_abr and a_abr2 are both > 3.5 m indicate an abrupt decline, while the normalised‐height‐decline or disruptedness metrics n_abr and n_abr2 indicate a continuous and undisrupted tree‐height decline.

**FIGURE A11:**
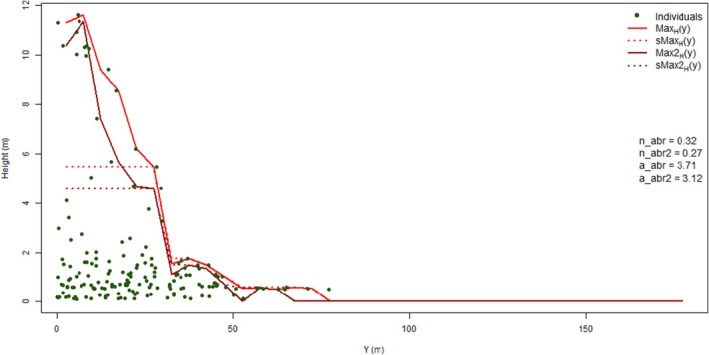
A simulated treeline where, from the top to the bottom of the transect, canopy height gradually ascends to nearly 2 m, and then abruptly increases to 4.5–5.5 m height. It is debatable whether or not this is an abrupt treeline. The abruptness indices *n*_abr_ (0.32) and *n*_abr2_ (0.27) mark it as ambiguous.

**FIGURE A12:**
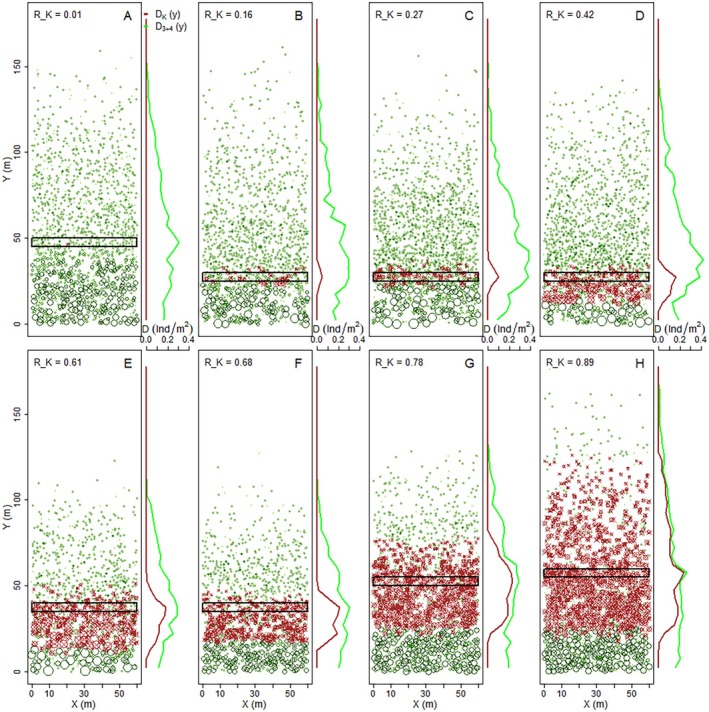
Top‐down view on four treelines from the simulated dataset. Symbol sizes correlate to square root of crown width, krummholz individuals are displayed in brown. The belt with highest krummholz density is framed. Although the width of the krummholz belt increases with R_K_ in these examples, that is not part of its definition.

The abruptness of a treeline ecotone within the framework presented by Bader et al. (2021) is considered as unrealised growth potential (due to lack of surviving individuals). As such, the proportion of unrealised growth becomes important in the quantification of the pattern. An ecotone where the trees decline to 10% of their potential height within the ecotone (i.e., at the bottom of the ecotone) before the canopy height breaks off, should be considered less abrupt than an ecotone where canopy height breaks off where trees still reach 50% of the potential height. In this sense, abruptness relates the height of the tallest individual that is missing in the ecotone with the height of the tallest individual that is there. This notion supports the use of the normalised metrics n_abr and n_abr2 over the absolute height difference metrics a_abr and a_abr2 (see also Figure A10). Naturally, this approach requires a robust definition and consensus of where the ecotone ends (Holtmeier 2009; Körner 2007, 1998), as canopy height is likely to increase further downhill (Holeksa et al. 2007; Stevens and Fox 1991). Within the modelled data, the parameters were selected to produce tree cover of at least 40% near the bottom of the simulated area to ensure a consistent ecotone border.

The absolute height differences, on the other hand, are less likely to be comparable between different sites, and do not take the context of the entire ecotone into account. However, these metrics do not require an exact definition of the ecotone borders, and therefore, the maximum canopy height reached within the ecotone. In our simulated dataset, there is a wide range of canopy heights at the bottom of the ecotone (largest individuals: 3.3–20 m), and considerably more variation than in the field data (9.7–15.5 m). However, these field data all represented the same species, so that a larger range can be expected when comparing treeline ecotones globally.

A difficulty with the approach used is the spatial resolution of the belts relative to the patterns studied. Although summarising forest properties in belts may be unavoidable, it also means a loss of information. Even with a resolution as fine as 5 m, it may occur that a drastic, steep decline, which would clearly be classified as abrupt in the field, could nevertheless be placed across two elevational belts if the canopy decline occurs across several individuals. This is the case at the Ordesa field site. This pattern drastically reduces the abruptness metrics, and makes it more difficult to identify an abrupt transition when only looking at the data vectors. One potential remedy here could be to calculate the abruptness measure multiple times, but shift the belts each time slightly (e.g., moving a 5‐m belt in 1‐m steps). The variability of these shifted metrics would allow to decide whether there is a steep but continuous (i.e., gradual) or a single‐step (i.e., abrupt) decline.

To capture the abruptness of the transition, we decided against fitting a function to the canopy height vectors *sMax*_*H*_(*y*) and *sMax2*_*H*_(*y*), like we did for tree density to define discreteness. At many treelines, canopy height continues to increase below the ecotone (Ameztegui et al. 2021; Holeksa et al. 2007), a pattern not captured by a logistic function (Bader et al. 2007).

#### 4.1.3 What Thresholds Delineate Abrupt and Gradual Treeline Forms?

The exact threshold to classify treelines into types of forms are necessarily arbitrary, but they can help to make clearer what we actually mean when we say that a treeline is “abrupt”, or “gradual”. In that sense, setting thresholds can be useful, while the exact thresholds will need to be agreed on by convention. It should be remembered that interpretation of the abruptness metrics and the optimal values for these threshold values are sensitive to the width of the bands used to calculate the maximum and second‐largest tree height. For example, an a_abr of 2 m indicates that the vegetation height decreased by 2 m over 5 m of horizontal distance, that is, 0.4 m per meter. With 10‐m bands, an a_abr of 2 m would mean 0.2 m per meter, that is, a more gradual decline. Our suggestion is to classify a treeline with less than 2 m decline per 5‐m elevational belt, that is, with at most 2 m at once and on average  < 0.4 m per meter (after smoothing and truncation) to be considered gradual. This is a bit less restrictive than the proposal of Bader et al. (2007), who consider declines from > 4 m‐tall forest to  < 1 m‐tall alpine vegetation over more than 10 m distance (i.e.,  < 0.3 m per meter) to be gradual. Those authors consider transitions from 4 m to 1 m over 4 m distance or less to be abrupt, which is just a bit more restrictive than our 3 m over 5 m. Based on our simulated dataset, we propose to use values of n_abr or n_abr2 of 0.25 as a threshold for gradual treelines, and 0.4 for abrupt ones. Both the absolute and the relative metrics leave an interim space which contains many ambiguous and intermediate examples. Bader et al. (2007) similarly use a third category between gradual and abrupt, which they call “moderately abrupt”.

Since in the tropical treeline ecotones studied by Bader et al. (2007) diffuse patterns were not observed, they did not differentiate between abruptness (height decline) and diffuseness (cover or density decline). They thus defined the gradualness based on height decline of a closed forest canopy. Although a closed tree canopy can also be heterogenous, the heterogeneity in the maximum‐tree‐height data is likely to be much higher in diffuse treelines, because towards the upper limit of a diffuse treeline, each elevational belt contains few trees. However, our truncating and smoothing procedures take care of this potential problem, and the meaning of abruptness is the same in discrete and diffuse treelines, showing whether growth (declining gradually) or additional processes limit the maximum elevation of tree occurrence. Therefore, we consider that the same thresholds for abrupt and gradual can be used, irrespective of ecotone discreteness.

### 4.2 Discrete to Diffuse

**FIGURE A13:**
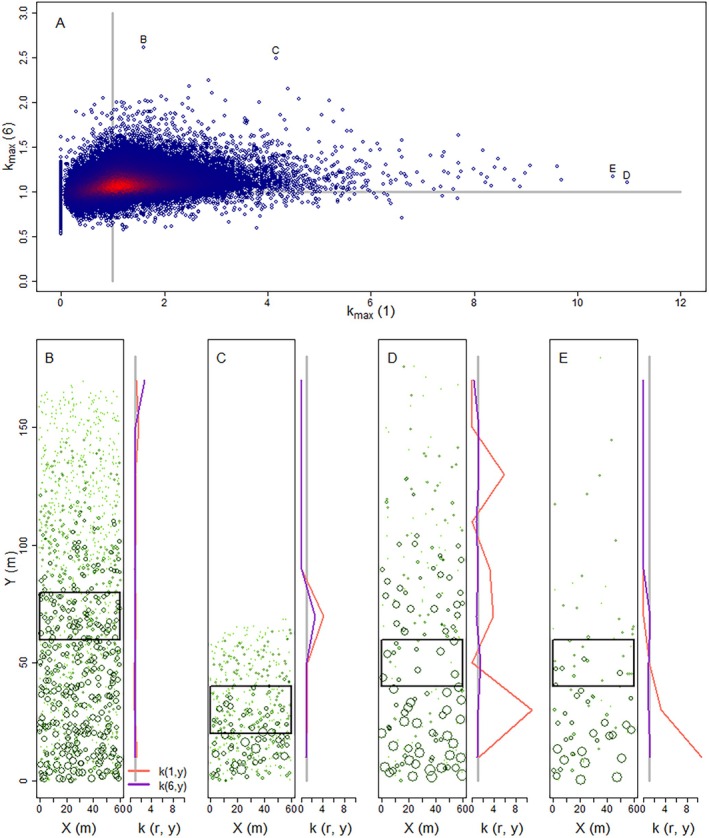
Assessing island‐type treelines. (A) Highest clustering in 1 m and 6 m radius in the simulated data. Grey lines indicate *k* = 1, that is, no clustering. The four simulated treelines with the strongest clustering are marked, and displayed in (B–E). In (B–E), individuals are displayed as circles, colours corresponding to layer. Although some aggregation of individuals is visible within the belts of strongest clustering (black frames), these are clearly not island‐type treelines.

For quantifying the discreteness of a treeline ecotone, both the density of individuals and tree cover could be used. After attempting to develop classification schemes for both we decided that tree cover is the most workable variable for defining treeline‐ecotone discreteness. Tree densities and point patterns more generally are very valuable for defining further pattern metrics, in particular the level of clustering (island formation or, the reverse, repulsion between individuals). In the model data used here, however, we found no indication of clustering (see Figure A13).

#### 4.2.1 Cover‐Based Quantification

Tree cover data are available in huge quantities in the form of remote‐sensing data, although at the spatial resolution needed for describing within‐ecotone patterns, data availability is still an issue, especially for remote mountain regions (Prakash et al., in prep). Still, remote sensing offers relatively easy access to tree‐cover information that can be used to quantify discreteness and other 2‐D spatial patterns—making data collection much easier than the collection of point‐pattern data in the field is. Turning remote‐sensing images into point pattern data is possible if tree crowns can be distinguished, which depends both on crown shapes and on the spatial resolution of the images. But although machine‐learning approaches are being developed fast and should make this task increasingly doable (e.g., Brandt et al. 2020), tree cover will always be easier to determine from the air than tree density. This, as well as the drawbacks of using tree density where trees are simultaneously declining in size, led us to define ecotone discreteness based on tree cover. For our field data, however, cover data was not available for some of the sites. We therefore estimated cover based on tree height, although this approach may introduce considerable uncertainty considering the large variation in crown geometries, especially for krummholz individuals. For that reason, crown diameter or other canopy‐cover estimates should be added in future sampling efforts.

The selected approach that uses a function fit requires a treeline that is straight and perpendicular to the elevational gradient. The discreteness of a site with strong horizontal sinuosity or high orientation index (Case and Hale 2015) will be underestimated by this approach. Sampling sites and transects should be therefore carefully selected, and this apply only applied to subsections with better aligned treelines, or adjusted elevational bands and subplot shapes to follow the edge of the forest rather than a straight simplification of the slope.

#### 4.2.2 What Thresholds Delineate Discrete and Diffuse Treelines?

Mountain forest can have open canopies even far below treeline, for example because of aridity or the proximity of boreal treeline (e.g., 40%–50% cover in Siberia, Hagedorn et al. 2014). Therefore, the discreteness metrics should also work with low *C*_max_ values. However, for low values of *C*_max_ the variation within the cover data vector may become high, rendering the fitted *s* parameter an unreliable predictor of discreteness (see Figure A7). Hence, in some dry or boreal mountains where tree cover is inherently low across elevations, it may be necessary to extend transects downhill to compensate for this increased variability. Generally, including a portion of forest extending below the ecotone is recommended for calculating robust *s* values, making sure that the entire transition in tree cover takes place within the sampled transect.

## 5 Conclusions

The treeline‐form metrics developed here are a first step towards a globally consistent synthesis of the patterns of treeline change. Further adjustments are envisioned based on increasing experience in their implementation at different field sites and for different models or model versions. To this end, further field or remote sensing data for real treelines are needed, especially to identify thresholds set to meaningfully delineate treeline forms. The classification suggested here is constrained to ecotones between 60 and 200 m in length, but the general method is applicable also to longer ecotones, where metrics can move outside the ranges found in our examples. The interpretation of one individual metric may occasionally depend on other metrics and additional ecotone characteristics. For instance, the abruptness may depend on the presence of krummholz. For discreteness, we believe we have found a coherent and comparable metric adequately quantifying the pattern. Abruptness remains a difficult pattern to quantify, but the four metrics proposed here may serve as basis of a debate on how the term could be defined quantitatively. As global synthesis becomes more and more important to address the challenges that global change poses for mountain regions (Price et al. 2022, pp. A‐3A6), methods and metrics that facilitate comparability are urgently needed.

## Author Contributions

**Lukas Flinspach:** data curation (lead), formal analysis (lead), methodology (lead), software (lead), writing – original draft (lead). **Thorsten Wiegand:** conceptualization (equal), funding acquisition (equal), methodology (supporting), supervision (equal), writing – review and editing (equal). **J. Julio Camarero:** data curation (supporting), methodology (supporting), writing – review and editing (supporting). **Enric Batllori:** data curation (supporting), methodology (supporting), writing – review and editing (supporting). **Emilia Gutiérrez:** data curation (supporting), methodology (supporting), writing – review and editing (supporting). **Maaike Y. Bader:** conceptualization (equal), funding acquisition (equal), methodology (supporting), supervision (equal), writing – review and editing (equal).

## Conflicts of Interest

The authors declare no conflicts of interest.

## Figures and Tables

**FIGURE A6:**
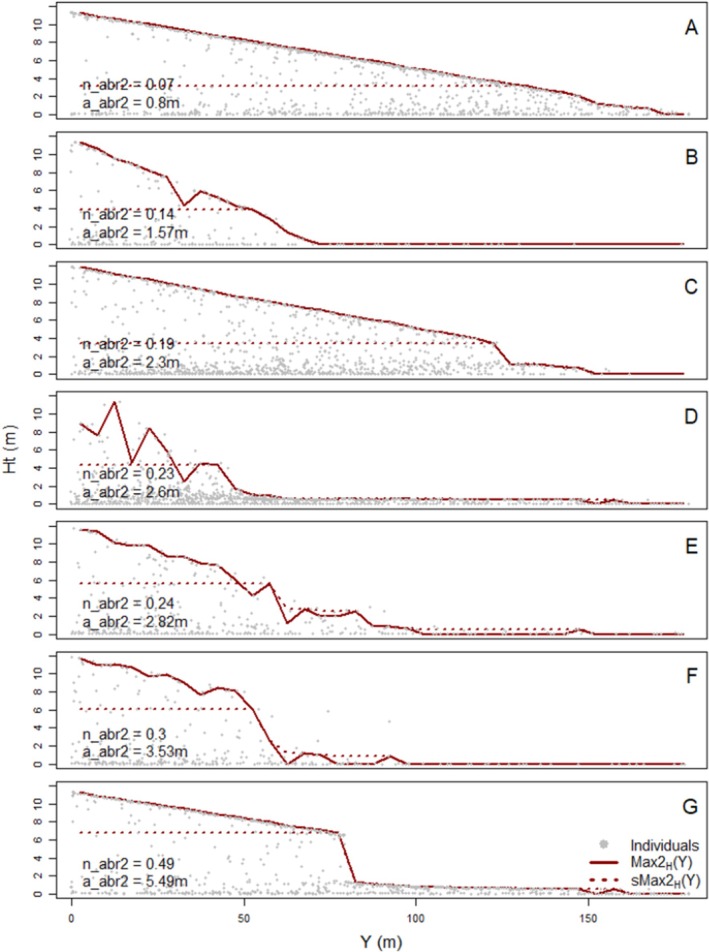
Gradient of abruptness, using different examples as in Figure 5 (main text). The n_abr2 metric ignores the tallest individual in each belt.

## Data Availability

Simulation data (i.e., parameterisation, summary statistics and treeline metrics of all simulations) are available on the zenodo platform, https://doi.org/10.5281/zenodo.15019445.

## Bottom Forest Parameterisation

To recreate realistic forests at the bottom of our simulated areas, and to thoroughly explore the parameter space of the model, we performed a simulation experiment with a latin hypercube sampling design (Stein 1987). We sampled 9330 parameterisations for 13 varied parameters. Parameter ranges were based on a previous simulation experiment, that is, parameter ranges were constricted to avoid simulations being cancelled due to producing average densities over 2 Ind/m^2^. Each parameterisation was run on a simulated world of 40 × 100 m, that is, consisting of two “elevational” belts and 20 subplot columns. Each parameterisation was run on 11 replicates for a total of 102,630 simulations. The elevational gradient parameters were set to 0, meaning there were no elevation effects in these simulations.

From each belt in each simulation, we calculated mean density of individuals in the protected layers 1&2 (D_12_), in layer 3 (D_3_) and in layer 4 (D_4_), the average height of individuals in layer 4 (H_4_), and the average age of individuals in layers 3 and 4 (A_34_). We did this by calculating each metric for each subplot, and subsequently calculating the belt‐wise averages. We did the same for the lower end of the four field sites from the Pyrenees (Sites Tesso and Ordesa: Camarero and Gutiérrez 2002; Capifonts and Portell: Batllori and Gutiérrez 2008).

## Field Data‐Based Rejection Filters

From the bottom forest parameterisations, we selected only those that consistently produced structurally realistic mountain forest. To achieve this, we used rejection filters (Hartig et al. 2011) based on field data. The minima and maxima of the filters were the based on the minima and maxima of the subplots in the field sites for the different summary statistics. The filters are presented in Table A1.

We only accepted parameterisations if the summary statistics of each replicate and in both belts always remained within these ranges. Additionally, we reduced the range of the parameter estab_mean to the maximum of 0.07. 121 bottom parameterisations were eventually selected and used in further simulation experiments.

**TABLE A1:** Rejection filters for the bottom parameterisations. These are based on subplots of the 4 field sites.

	D_3+4_ (Ind/m^2^)	D_1+2_ (Ind/m^2^)	H_4_ (m)	A_3+4_ (years)
Min	0	0	2.9	21
Max	0.25	0.12	12.4	136.4

## Gradient Run Simulation Experiments

We conducted a total of 11 simulation experiments. In each, we selected a specific combination of first‐level gradient (growth, seedling survival, dieback) and optional submodels (facilitation, dieback protection), that is, we varied the elevation‐based parameters gg_12_, gg_34_, sg_1_, dg as well as sZ and dZ, which had previously been set to 0. We evenly sampled gradient parameter range using Latin hypercube sampling. For the growth gradients, we imposed the restriction of gg34 > gg12, that is, growth rates decline faster with elevation outside the protected layer. We combined each sampled gradient parameter (combination) with each bottom parameterization, and ran every resulting new parameterization in several replicates, see Table A2.

**TABLE A2:** Design of the simulation experiments. Each sampled gradient parameter (combination) was paired with each selected bottom parameterization, see previous section.

Exp. name (abbr.)	Limiting gradients and processes	Varied parameters	# of samples	# of new parameterisations	# of simulations
gr	Growth only	gg_12_, gg_34_	20	2420	14,520
su	Seedling survival only	sg_1_	20	2420	14,520
suf	Survival and facilitation	sg_1_, sZ	20	2420	18,150
Gr‐su	Growth and survival	gg_12_, gg_34_, sg_1_	25	3025	7260
Gr‐suf	Growth and survival with facilitation	gg_12_, gg_34_, sg_1_, sZ	30	3630	14,520
di	Dieback only	dg	10	1210	14,520
Gr‐di	Growth and dieback	gg_12_, gg_34_, dg	20	2420	21,780
Su‐di	Survival and dieback	sg_1_, dg	20	2420	14,520
Gr‐su‐di	All three	gg_12_, gg_34_, sg_1_, dg	30	3630	21,780
Gr‐di‐suf	All three with facilitation	gg_12_, gg_34_, sg_1_, dg, sZ	30	3630	21,780
Gr‐dip‐suf	All three with facilitation and protection	gg_12_, gg_34_, sg_1_, dg, sZ, dZ	30	3630	21,780
	Total		255	30,855	185,130

## Treeline Acceptance Criteria

As a consequence of the varied intensity of the limiting gradients, many of the simulations did not produce a treeline ecotone, generally because the imposed gradients did not sufficiently restrict tree growth at the top of the simulated transect. The criteria to remove unsuitable simulations used here were the following:

- D_3+4_ > 0.01 individuals/m^2^ in the lowest 20 m of the simulated area (0 m < *y* < 20 m)
- D_3_ < 0.01 individuals/m^2^ in the topmost 20 m (160 m < *y* < 180 m) (see Wiegand et al. 2006)
- D_4_ = 0 in the topmost 20 m of the simulated area.

In this work, we focus on parameterizations where all of the n_rep_ = 6 replicate simulations did produce a treeline ecotone meeting the above criteria.

## Comparison of Different Canopy Data Vectors

We tested different summary statistics vectors to describe canopy height to find summary statistics that are both robust to low individual density and high variation, which may be common at treelines, but also not biased by low stature individuals in the undergrowth. In each subplot, we selected the tallest individual above 0.5 m (“subplot canopy height”), and the second tallest (“subplot subcanopy height”). For the data vectors aggregating these subplot metrics across the elevational gradient, we tested the selecting the tallest subplot canopy height in each belt (Max_H_ (y), see Figure A5), the second‐tallest subplot canopy height (Max2_H_ (y)), and the average of subplot canopy height (Mn_H_ (Y) in Figure A5). To complement Max2_H_, we also tested the highest value of the subplot subcanopy height in each belt (SubMax_H_ (Y)). We found Mn_H_ (Y) too strongly affected by small, not fully grown individuals, and not sufficiently able to track the height of the tallest individuals on visual inspection, see Figure A5.

We found SubMax_H_ (Y) to have more missing values than Max2_H_ (y), and, for most parameterisations, no improvement in variation compared to Max2_H_ (y). For these reasons, we used only Max_H_ (y) and Max2_H_ (y) for quantification and classification of treelines.

## Subplot Cover Estimation

Tree cover is calculated by subdividing the simulated, respectively field area, into cells of 1 × 1 m. Subsequently, all individuals above 0.1 m height are then divided into large and small trees based on crown diameter. Crowns are assumed to be circular. In the field data, where we had no crown radius available, we assume it to be 25% of tree height. Tree crowns larger in diameter than the cell diagonal are assumed to cover 100% of all cells if the centre of the cells are directly underneath the crown, that is, within crown radius of the tree position. For smaller crowns, their area is projected directly onto the cell (i.e., a crown half the area of a grid cell covers 50% of the cell the individual is located on). For each cell, cover is capped at 100%.

Subsequently, for each subplot (of 5 × 5 m), the average tree cover of all cells is calculated. This subplot value is used to fit the cover function (Equation 5 in the main text). We considered, but ultimately rejected, the use of a belt‐wise average of subplot cover for the fit.

## Density‐Based Classification of Discreteness

An alternative choice of data vector to quantify discreteness of a treeline is tree density. We fitted the same logistic function described in the main text (Equation 5) to density data vectors. Note that in our model, trees are categorised into different height classes, whose density is calculated separately. In addition to the logistic function, we also fitted a unimodal function of the following form to tree density (in layers 2, 3 and 4):

(E1)
$$D\left(y\right)=\frac{D_{\max}}{e^{\left(\frac{{\left(y-y_{p}\right)}^{2}}{w}\right)}}$$

With the parameters *D*_*Max*_ (peak density), *y*_p_ (location of the peak along the *y*‐axis) and *w* (width of the bell curve). This unimodal function was intended to capture two patterns: As density and crown radius are inversely related, and crown radius should decline with elevation, we expect a density spike or peak near the top of a discrete treeline ecotone. This spike should ideally be quantified by the max‐parameter of the function. Additionally, the width parameter *w* quantifying the wideness of the wide bell shape may provide additional information on the diffuseness of the treeline.

Due to the difficulty in capturing the patterns resulting from discrete treelines in density data (see Figure 4 in the main text), we did not use the density data vectors for classification here.

## Krummholz Metric

Besides the abruptness and discreteness metrics presented in the main text, we also developed a metric to describe the prevalence of krummholz at treelines.

To this end, we first need to identify and label krummholz individuals. Labelling may be performed quantitatively (e.g., continuous deformation on a scale of 0%–100%, categorical “none”, “mild”, “severe”, or qualitatively with a binary distinction). In both the model and the field data, krummholz individuals were labelled qualitatively. We consider this approach both more practical during sampling, and more robust against subjective bias.

In the collection of new field data, it is recommended to also add a krummholz label. In an alternative approach, applicable to existing field data where such a label is lacking, crown width could be related to tree height, assuming biomass loss affects height growth more severely than lateral growth. Krummholz density data vectors D_K_ (y) were calculated on subplot basis, as described in the main text for tree densities, and thus follow the same approach as Wiegand et al. (2006).

As the metric for the importance of krummholz at the treeline we used the relative density of krummholz in the specific elevational belt *y*_K_ where krummholz density is highest, that is, the ratio:

(E2)
$$R_{K}=\frac{D_{K}\left(y_{K}\right)}{D_{3+4}\left(y_{K}\right)},\text{with}\;D_{K}\left(y_{K}\right)=\max\left[D_{K}\left(y\right),y\right]$$

A high value of this metric indicates a large prevalence of krummholz. Note that this metric does not require the krummholz belt to be large relative to the ecotone length, which makes it more applicable to treelines of different length.

To avoid distortion from random krummholz individuals, we require a minimum density of krummholz individuals to consider this metric. We used a minimum density of 0.05 individuals/m^2^ per elevational belt to consider it as a krummholz treeline in our model simulations. This value was based on the Pyrenean Odessa field site, where krummholz density ranges between 0.11 and 0.58 individuals/m^2^ within the krummholz belt (Camarero and Gutiérrez 2002). We chose half that value to accommodate also more diffuse treelines.

Figure A12 displays a range of simulated treelines with increasing “krummholzness” index R_K_. We propose to classify a treeline with an R_K_ index > 0.5 as a krummholz treeline (Figure A12).

## Islandness Metric

For discovering spatial clustering of trees indicative of island‐type tree clusters, we used the transformation *k*(*r*) = *K*(*r*)/(π *r* ^2^) of Ripley's *K*‐function *K*(*r*) (Wiegand and Moloney 2004). This function counts the mean number of other individuals within distance *r* of each individual, and divides it by its expectation of the null model of complete spatial randomness (CSR). It is basically a cumulative version of the pair‐correlation function *g*(*r*). The function *k*(*r*) indicates clustering if *k*(*r*) > 1 or overdispersion if *k*(*r*) < 1. Due to the scale of our dataset, and the low densities resulting from the elevational gradients within our simulations, we were forced to use a few simplifications.

First, the metric is sensitive to low densities (Batllori et al. 2010; Wiegand and Moloney 2004). Therefore, we used larger belts here (20 m width instead of 5 m), and calculated the metric only if the density of individuals exceeded 0.2 individuals/m^2^. Additionally, rather than calculating the *k*(*r*)‐function for a range of radii, and thereby adding an additional spatial axis to the existing elevational axis, we focused on two fixed radii. We selected the radii of *r* = 1 m and *r* = 6 m. These radii were chosen in correspondence with field experts and selected to match typical tree island dimensions.

We thus obtained for each belt *y* = 10, 30, 50 m, … the two values *k*(*r* = 1, *y*) and *k*(*r* = 6, *y*). To quantify the “islandness” of an individual transect or simulation, we used the maximum value *k*_max_(1), *k*_max_(6) defined as:

(E3)
$$k_{\max}\left(1\right)=\max\;\left[k\left(r=1,y\right),y\right])$$

(E4)
$$k_{\max}\left(6\right)=\max\;\left[k\left(r=6,y\right),y\right])$$

This approach assumes that, due to the lower overall density in the “island” part of the ecotone, the k metrics should always reach their maxima in this part, even if there is also clustering in the forest below. NA values, that is, belts with low densities, are ignored here.

However, our simulated data do only show low levels of clustering and no island‐type treeline emerged. Visual inspection of the simulated area of four simulations with the strongest clustering according to the *k*_max_ –metrics does not provide indication of islands in the simulations (see Figure A13). For all four simulations, the other replicates with identical parameterization were less conspicuous.

**TABLE A3:** Extended description of the pattern indices.

Krummholzness	*R*_K_	Relative density of krummholz in belt of highest krummholz density
Islandness	*k*_max_(1)	Highest value of *k*(*r* = 1 m, *y*)
	*k*_max_(6)	Highest value of *k*(*r* = 6 m, *y*)
